# Multistep assembly of DNA condensation clusters by SMC

**DOI:** 10.1038/ncomms10200

**Published:** 2016-01-04

**Authors:** HyeongJun Kim, Joseph J. Loparo

**Affiliations:** 1 Department of Biological Chemistry and Molecular Pharmacology, Harvard Medical School, Boston, Massachusetts 02115, USA

## Abstract

SMC (structural maintenance of chromosomes) family members play essential roles in chromosome condensation, sister chromatid cohesion and DNA repair. It remains unclear how SMCs structure chromosomes and how their mechanochemical cycle regulates their interactions with DNA. Here we used single-molecule fluorescence microscopy to visualize how *Bacillus subtilis* SMC (BsSMC) interacts with flow-stretched DNAs. We report that BsSMC can slide on DNA, switching between static binding and diffusion. At higher concentrations, BsSMCs form clusters that condense DNA in a weakly ATP-dependent manner. ATP increases the apparent cooperativity of DNA condensation, demonstrating that BsSMC can interact cooperatively through their ATPase head domains. Consistent with these results, ATPase mutants compact DNA more slowly than wild-type BsSMC in the presence of ATP. Our results suggest that transiently static BsSMC molecules can nucleate the formation of clusters that act to locally condense the chromosome while forming long-range DNA bridges.

## Abstract (web summary)

The Structural Maintenance of Chromosomes (SMC) proteins are essential for chromosome condensation, cohesion and DNA repair. Here the authors use single molecule imaging to visualise how *Bacillus subtilis* SMC interacts with and condenses DNA.

## Graphical abstract

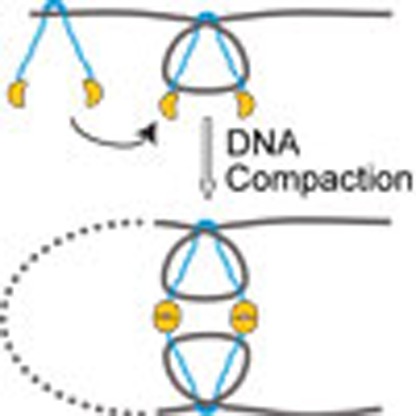

Across kingdoms of life, organisms are faced with the challenge of packaging their DNA within cells whose dimensions are orders of magnitude smaller than the linear length of their genetic material. DNA-binding proteins that bend, wrap or bridge DNAs help to condense DNA to package it within the cellular volume^1^,^2^,^3^. SMC (structural maintenance of chromosome) proteins are a highly conserved protein family that play critical, yet largely mysterious roles in chromosome organization^4^,^5^,^6^. Eukaryotes have six SMC paralogues: the SMC1–SMC3 cohesin complex establishes sister chromatid cohesion^7^, the SMC2–SMC4 condensin complex plays a crucial role in compacting and segregating chromosomes^8^ and the SMC5–SMC6 complex tethers DNA ends during recombinational DNA repair and other damage response pathways^9^. Most prokaryotes have a single *smc* gene, and its homodimer constitutes the core SMC complex. Bacteria, however, do possess a number of SMC-like proteins including a functional homologue of SMC called MukB (and its binding partners MukE and MukF) found in some γ-proteobacteria, and others such as SbcC, RecN and SbcC2, that are involved in DNA repair^10^.

SMC proteins are large (1,000–1,300 amino acids, ∼50 nm in length) and characterized by a unique structure (Fig. 1a)^11^. The N and C termini contain nucleotide-binding motifs known as Walker A and Walker B motifs, respectively. The middle of the SMC sequence forms a globular hinge domain, which is flanked by coiled-coil regions. SMC monomers fold around the hinge domain, forming intramolecular anti-parallel coiled-coil arms^12^ and an ATPase head domain that contains the N and C termini^13^. SMC monomers interact through their hinge domains^12^ to form V-shaped dimers^14^. Binding of ATP within the ATPase head domains leads to their association, where ATP is sandwiched between the Walker A/B motifs of one monomer and the C-motif (or signature motif) from the other^15^,^16^. Interaction with DNA stimulates hydrolysis of ATP within the SMC head domains, leading to their disengagement^15^,^17^,^18^.

Non-SMC subunits are essential components of the SMC complex. The conserved kleisin protein interacts with both SMC monomers and binds a second non-SMC component that varies among species. Together, these SMC accessory factors modulate both the ATPase activity and DNA-binding affinity of SMC^11^,^19^,^20^,^21^,^22^. The prokaryotic SMC interacts with the kleisin protein ScpA (segregation and condensation protein A) and a second component ScpB asymmetrically in a ratio of 2:1:2 (refs 23, 24).

Fluorescent protein fusions to bacterial SMCs demonstrate that they form bipolar foci near replication origins. In *B. subtillis* and *Streptococcus pneumoniae*, SMC is recruited in part by the nucleoid-associated protein ParB, which binds to *parS* sites near the origin region^25^,^26^,^27^. Deletion of the bacterial *smc* or either of its non-SMC subunits leads to defects in origin resolution, abnormal nucleoid structure, a significant increase in the percentage of anucleate cells and other phenotypes consistent with the SMC complex playing a vital role in chromosome organization and segregation^28^,^29^. Yet, the mechanism by which origin-proximal SMCs facilitate segregation of the chromosome remains unclear.

Understanding how SMCs structure chromosomes requires a molecular description of how SMC interacts with DNA and how these interactions are coupled with the ATPase cycle of SMC. SMCs may act as a ring, encircling two DNA segments without making strong contacts with DNA^30^,^31^. While this ‘topological' model of SMC association with DNA is appealing for cohesin, it remains unclear how such a mode of interaction would be effective for condensin. DNA loops trapped within a diffusible condensin ring should be susceptible to unravelling unless the DNA is topologically entrapped within SMC rings^31^. Structural studies have suggested much stronger interactions between SMCs and DNA, including the bending (or wrapping) of DNA around the head domains of SMC^32^ potentially mediated through a positively charged patch on top of the head domains^15^,^16^,^33^. DNA bending is consistent with the observation that bacterial SMCs and condensins constrain positive supercoils^34^,^35^. While a model of DNA bending by SMC provides a direct mechanism by which individual SMCs might organize the chromosome, the small number of SMCs per cell (5–10 kb per SMC complex)^36^,^37^ suggests that it is unlikely to be the only mechanism by which SMC influences chromosome organization.

Single-molecule methods are a powerful tool to address how SMCs interact with DNA and change its conformation. Prior studies utilizing magnetic tweezers to weakly stretch DNA demonstrated that SMC family members condense and bridge DNAs *in vitro*^38^,^39^,^40^,^41^. Yet, these approaches are limited in that they are unable to visualize SMCs directly. To address this limitation, we have combined DNA flow stretching with single-molecule fluorescence imaging of the prokaryotic model SMC from *Bacillus subtilis* (BsSMC). This approach has revealed a sequence of SMC–DNA interactions that lead to the assembly of DNA condensation clusters. We show that individual SMCs bind DNA, alternating between static binding and one-dimensional (1D) diffusion. Continued association of additional BsSMCs leads to the formation of complexes along DNA. These BsSMC clusters reshape DNAs in a weakly ATP-dependent and cooperative manner, by both bending DNA locally and bridging DNA loops over long distances. Collectively, these results show that SMCs have varied interactions with DNA that evolve as they assemble into multimeric complexes.

## Results

### BsSMCs switch between diffusion and static binding on DNA

**Figure 1:**
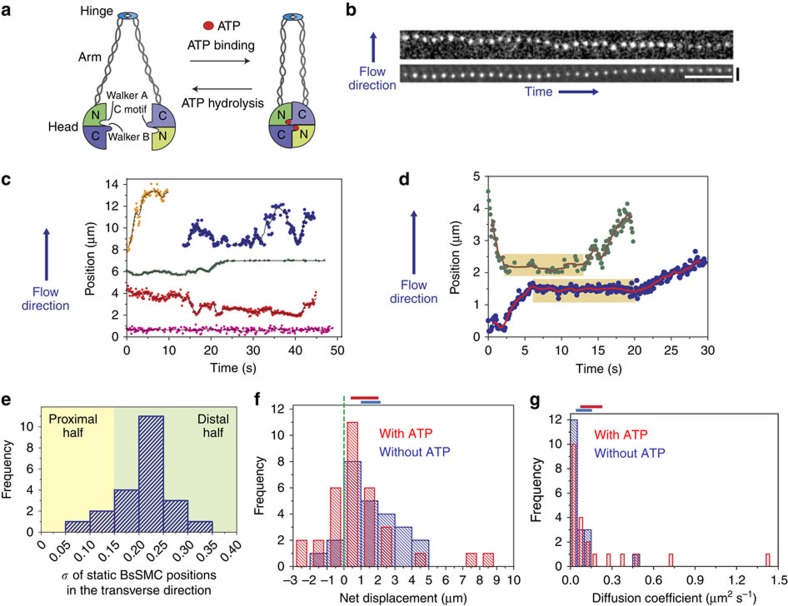
Characterization of BsSMC sliding along DNA. (**a**) Cartoon of the BsSMC dimer. The Walker A motif resides on the N-terminal half of the head, while Walker B and C motifs are located at the C terminal part of the head. Not drawn to scale. (**b**) Snapshots from two different DNAs showing diffusion of labelled wild-type BsSMC (∼2 nM) on a flow-stretched DNA in the presence of 1 mM ATP. White and black scale bars, 5 s and 3 μm, respectively. (**c**) Trajectories of BsSMC in the presence of 1 mM ATP. Different events were denoted with different colours, and the starting point of each event was arbitrarily set for visualization on the same plot. (**d**) Two trajectories of wild-type BsSMC showing transitions between static association and 1D diffusion in the presence of 1 mM ATP. Light-brown boxes represent periods of static binding as defined by the criteria described in the text. (**e**) A histogram of s.d. of statically bound BsSMC position in the transverse direction (*n*=22). Light-yellow and green colours denote approximate regions of the proximal and distal halves of the λ-DNA, respectively. This distinction was determined based on the characterization of transverse fluctuation along the DNA length (Supplementary Fig. 1e). (**f**) Histograms of net displacement. Positive values represent net displacement towards the free end of the DNA. A dotted vertical green line represents zero net displacement. Horizontal blue (*n*=25) and red (*n*=33) bars in the histogram represent data without and with ATP, respectively (*P*>0.2 by *t*-test). The horizontal blue (without ATP) and red (with ATP) lines on the top of the graph represent 95% confidence intervals obtained by bootstrapping analyses. (**g**) Histograms for diffusion coefficients of wild-type BsSMC. *n*=19 for blue bars, *n*=21 for red bars (*P*>0.2 by *t*-test). The horizontal blue (without ATP) and red (with ATP) lines on the top of the graph correspond to 95% confidence intervals from bootstrapping analysis.

Using single-molecule fluorescence imaging, we monitored the interaction of fluorescently labelled SMC dimers with flow-stretched DNA molecules. We labelled non-specific lysines of BsSMC using Cy3 NHS ester under conditions that resulted in ∼50% labelling of SMC monomers (or roughly one Cy3 per SMC dimer; Supplementary Fig. 1a). This labelling did not affect the ability of BsSMC to compact DNA (see Methods). Bacteriophage λ genomic DNAs were tethered at one end to a polyethylene glycol (PEG)-passivated microfluidic flow cell via a biotin–neutravidin interaction. The flow of buffer through this flow cell resulted in the extension of the DNAs. Upon addition of a 1–2 nM labelled BsSMC, a fluorescent focus appeared on a subset of individual DNAs. Given the low protein concentration, most DNAs (>90%) had no labelled BsSMCs bound, and DNAs were not observed with more than one spot at a time. Furthermore, an integrated intensity comparison of BsSMC on DNA and nonspecifically surface-bound BsSMC estimated that most of BsSMCs on DNA, if not all, bound as single dimers (Supplementary Fig. 1b; see Methods). Movies of BsSMC dimers were recorded and trajectories of BsSMC position over time were obtained by fitting two-dimensional Gaussian functions to the foci of each frame of the movie. Trajectories of nonspecifically surface-bound BsSMC were also obtained to confirm that stage drift did not contribute significantly to the motion of diffusing BsSMCs on DNA (Supplementary Fig. 1c). Example snapshots and trajectories of BsSMC motion on individual DNAs can be found in Fig. 1b,c.

Trajectories of BsSMC motion could be classified into three categories as follows: (1) 1D diffusion along DNA for the entire trajectory; (2) static association with DNA; and (3) transitions between 1D diffusion and static association (Fig. 1d). BsSMCs are never truly ‘static' due to fluctuations of the underlying DNA. We characterized these DNA fluctuations by labelling DNA site specifically at five different loci with catalytically inactive EcoRI conjugated with quantum dots, a technique we call DNA motion capture^42^,^43^. The magnitude of both longitudinal and transverse DNA fluctuations increased with increasing distance from the tether point (Supplementary Fig. 1d,e). Analysis of these trajectories showed that quantum dot motion became rapidly bounded; a plot of the mean-square displacement (MSD) reached a plateau in under 0.5 s. To distinguish between the static and mobile segments of the BsSMC trajectories, we calculated MSDs of seemingly static BsSMCs along DNA. If a graph of MSD versus time showed bounded motion (reaching a plateau within 0.5 s) or if the MSD was <0.2 μm^2^, then the spot was classified as being statically bound. Using these criteria, we found that for trajectories that showed both static and mobile behaviours, 41.5±5.7% and 51.3±5.7% (means±s.e.m.) of the duration of the trajectory were in the static state in the absence and presence of ATP, respectively. However, comparisons of these duration distributions using a *t*-test were not statistically significant (*P*>0.1).

We estimated the position of statically bound BsSMCs along DNA by comparing the s.d. of transverse fluctuations of BsSMC foci with the values obtained for the site specifically bound quantum dots (see Fig. 1e and compare with Supplementary Fig. 1e). A histogram of the s.d. of position fluctuations for static BsSMC trajectories was quite broad and is consistent with BsSMC being able to statically associate throughout the DNA substrate. The magnitude of the s.d. of the position of nonspecifically surface-bound BsSMCs was substantially lower than the values we obtained for BsSMCs statically bound to DNA (Supplementary Fig. 1f). This strongly suggests that these static, DNA-bound BsSMCs and the underlying DNA are not nonspecifically bound to the glass surface. Static BsSMC localizations were weighted to the second half of the DNA molecule likely due to bias in diffusion introduced by flow (described below). Consistent with this proposal, we observed that static BsSMC molecules that persisted until the end of the experiment were often at or near the free end of DNA as observed by addition of the DNA staining dye SYTOX Orange (Supplementary Fig. 1g).

For the mobile segments of the BsSMC trajectories, we first asked whether the buffer flow biased the motion of the protein towards the free end of DNA. For each trajectory, we calculated a net displacement that is equal to the position difference between when BsSMC appeared and disappeared on DNA. Overall, 88 and 70% of the events showed positive net displacements (moving towards the free DNA end) in the absence and presence of ATP, respectively (Fig. 1f), although the average displacement was quite small (1.56±0.31 μm without ATP, 1.01±0.41 μm with ATP; mean±s.e.m). The presence of ATP did not generate a statistically significant difference in the net displacement histograms (*P*>0.2 by *t*-test, 95% confidence interval from bootstrapping analysis with 2,000 resamplings overlaps. (0.99, 2.15) μm without ATP, (0.40, 2.03) μm with ATP). Next, we calculated the drift of each diffusing BsSMC, defined as the net displacement of each trajectory divided by the time period. They are 97±35 (mean±s.e.m.; without ATP) and 96±61 nm s^−1^ (with ATP), respectively (Supplementary Fig. 1h; *P*>0.2 by *t*-test. 95% confidence interval from bootstrapping analysis: (50.04, 212.26) nm s^−1^ without ATP, (−9.85, 228.78) nm s^−1^ with ATP). To further characterize the bias from flow, we concatenated all mobile trajectories as if they were part of a single trajectory^44^ and determined the average drift velocity that was 0.07 and 0.04 nm s^−1^ in the presence and absence of ATP, respectively. The orders of magnitude difference between the individual drift velocity and the concatenated drift velocity further demonstrate that the motion of BsSMC is only slightly biased by flow.

To calculate the diffusion coefficient of BsSMC, we first corrected the individual position trajectories of BsSMC molecules for bias due to flow (see Methods). With these corrected position trajectories, we calculated MSDs that were well fit by a linear regression (Pearson's coefficient >0.9). The resulting mean diffusion coefficients were 0.070 μm^2^ s^−1^ (0.61 × 10^6^ bp^2^ s^−1^) in the absence of ATP and 0.131 μm^2^ s^−1^ (1.13 × 10^6^ bp^2^ s^−1^) in the presence of ATP (Fig. 1g). The mean value difference is again not statistically significant, since the *P* value from a *t*-test comparison of the distributions is >0.2 and the 95% confidence intervals of mean diffusion coefficients from bootstrapping analysis overlapped significantly with each other (without ATP: (0.0390, 0.155) μm^2^ s^−1^, with ATP: (0.071, 0.230) μm^2^ s^−1^; see Methods). These diffusion coefficients are comparable to other DNA-binding proteins known to slide along DNA, including ParB (Spo0J), PCNA and full-length p53 (refs 43, 44, 45, 46). In sum, the lack of ATP dependence on BsSMC motion on DNA, along with the linear MSD plots we observe, is consistent with Brownian diffusion instead of an active translocation process.

### BsSMC molecules form clusters that compact DNA

Bacterial SMCs form foci in cells^22^,^25^,^26^,^27^,^47^. We asked whether this behaviour could be reconstituted using our labelled SMC. We performed flow-stretching experiments with a Cy3-labelled BsSMC concentration of 120 nM in both the presence and absence of ATP. To investigate whether these concentrations of BsSMC could condense stretched DNAs, we attached a spectrally distinct quantum dot to the free end of λ-DNA so that we could simultaneously correlate DNA length changes with protein binding (see Methods for details of quantification).

**Figure 2:**
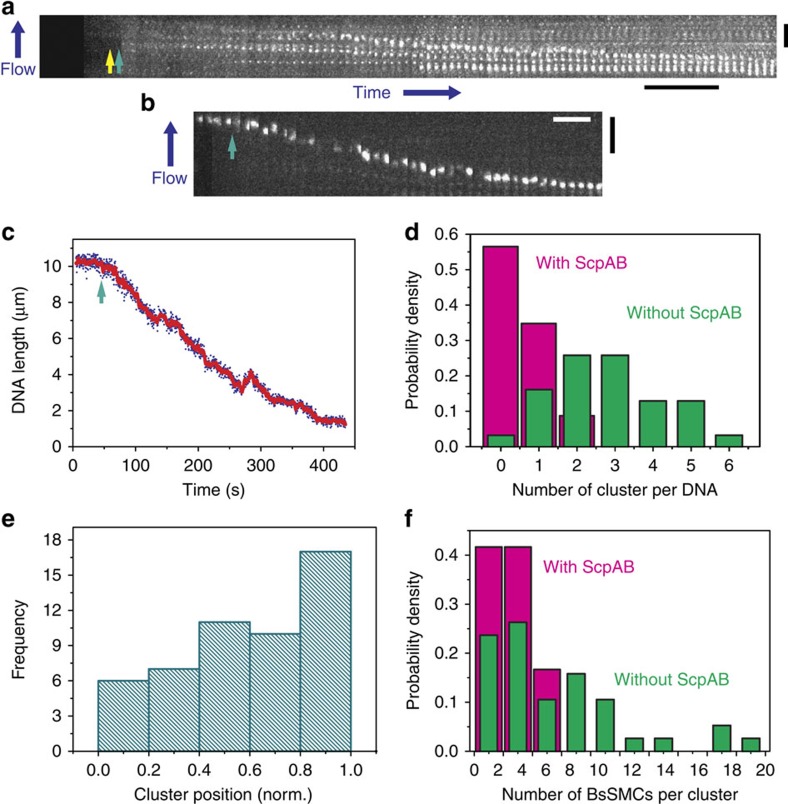
BsSMC molecules can form clusters on DNA. (**a**) Example kymograph depicting BsSMC cluster formation in the absence of ATP. The appearance of the first visible cluster is marked with a yellow arrow, and the onset of compaction, identified by tracking a DNA end-labelled quantum dot (shown in **b** and **c**), is marked with a green arrow. Scale bars, 40 s, 5 μm (for both panels). (**b**,**c**) Kymogram and trajectory of the spectrally distinct quantum dot attached at the end of DNA shown in **a**. Again, the starting point of compaction is marked with a green arrow. (**d**) Number of clusters on DNA in both the presence (magenta, *n*=23) and absence (green, *n*=31) of ScpAB. [BsSMC]=120 nM, [ATP]=1 mM, ([ScpA]=480 nM, [ScpB]=960 nM), 5 min after sample loading. (**e**) Estimated cluster positions normalized with respect to tether point (position: 0.0) and the end of DNA (position: 1.0) in the presence of ATP and absence of ScpAB. (**f**) Estimated number of BsSMC within a cluster in both the presence (magenta, *n*=12) and absence (green, *n*=38) of ScpAB. [ATP]=1 mM.

Even with the high concentration (120 nM) of labelled BsSMC in the flow cell, we observed the formation of BsSMC clusters whose signals stood out of the background on flow-stretched DNAs in both the presence and absence of ATP (Fig. 2a; Supplementary Fig. 2a). This pattern of binding to DNA is qualitatively different from that of *B. subtilis* ParB (Spo0J), which binds uniformly to non-specific DNA^43^. After the appearance of the first cluster, the DNA-end-bound quantum dot was observed to move towards the DNA tether point and BsSMC clusters moved closer together, consistent with BsSMC clusters compacting DNA (Fig. 2a–c; Supplementary Fig. 2a). We also observed that clusters could merge together as DNA condensation brought clusters next to each other (Supplementary Fig. 2a). Given that DNA condensation occurred nearly continuously and that the appearance of new clusters was relatively infrequent, our data suggest that existing clusters, and not just initial formation, contribute to DNA compaction. This condensation may result from the recruitment of additional BsSMCs to clusters; consistent with this idea, we found that the intensities of clusters increased over time (Supplementary Fig. 2b,c).

Cluster formation occurred robustly with 97% of DNAs displaying at least one cluster after 5** **min of protein loading (Fig. 2d). These clusters appeared throughout the DNA (Fig. 2e), although their position was somewhat biased towards the free end (see Methods). Preincubation of SMC with four- and eightfold molar excesses of ScpA and ScpB, respectively, reduced the average number of clusters on DNA (0.5±0.7 with ScpAB, 2.8±1.5 without ScpAB; mean±s.d.; Fig. 2d). This difference was further supported by bootstrapping analyses that showed that the 95% confidence intervals ((0.304, 0.826) with ScpAB, (2.323, 3.354) without ScpAB) were non-overlapping. Clusters were also dimmer in the presence of ScpAB. By calibrating to the intensity of individual BsSMCs nonspecifically stuck to the coverslip surface, we related cluster brightness to SMC copy number within a cluster. At a time point of 5 min after initiating the flow of protein into the flow cell, we found on average 2.72 BsSMCs per cluster with ScpAB and 5.60 BsSMCs per cluster without ScpAB (95% confidence interval from bootstrapping analyses: (1.846, 3.753) with ScpAB, (4.279, 7.302) without ScpAB; Fig. 2f).

### BsSMCs compact DNA by both bending and bridging

To further investigate the molecular mechanisms of BsSMC-mediated DNA compaction, we employed the DNA motion capture assay. We previously demonstrated how DNA motion capture can be used to distinguish between DNA bending (or wrapping) and bridging^43^. Given the differential tension along a flow-stretched DNA and the sensitivity of DNA loop formation to applied force, DNA-bridging proteins preferentially compact DNAs at the free end where the tension is the lowest. On the other hand, DNA-bending proteins, such as the HU homologue HBsu, compact stretched DNAs globally, as they are not as sensitive to the variation in DNA tension found along the flow-stretched DNA. In contrast to bridging proteins, bending proteins do not compact flow-stretched DNAs to the tether point as there are a finite number of DNA-bending sites available and each bending event reduces the DNA length by only a modest fraction^48^,^49^,^50^.

**Figure 3:**
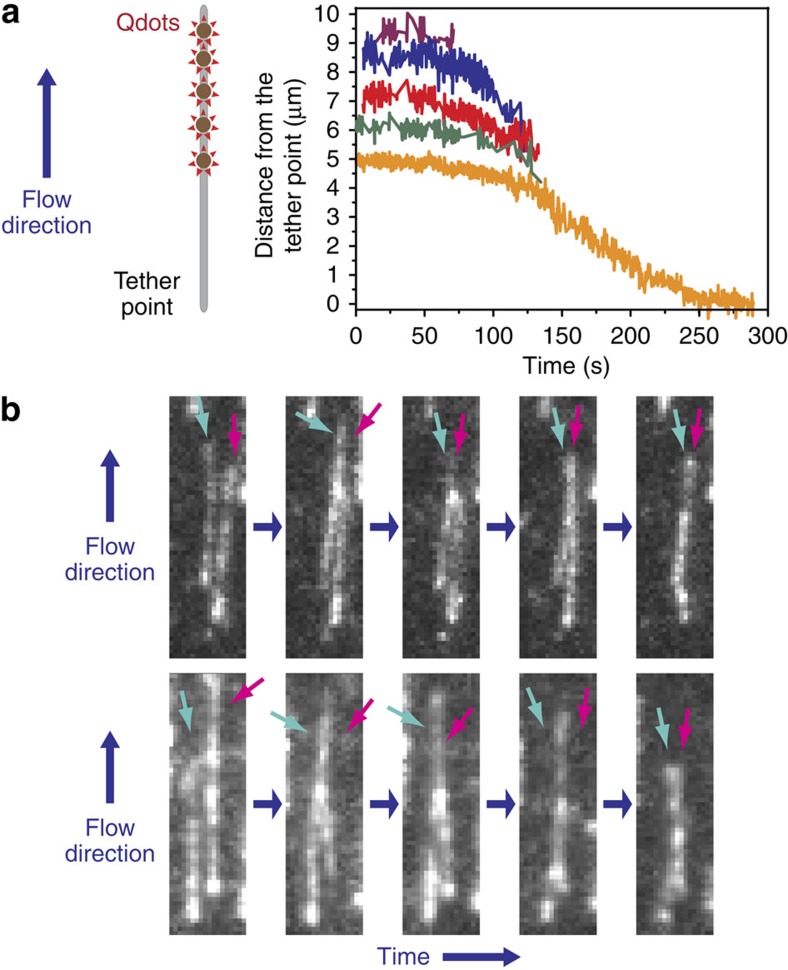
BsSMC shows mixed compaction modes. (**a**) A representative trajectory from a DNA motion capture experiment showing DNA compaction mediated by wild-type BsSMC with 1 mM ATP. The position of each quantum dot is shown by a different coloured line. DNA compaction eventually makes it impossible to resolve adjacent quantum dots. Compaction completes at the tether point. (**b**) Two representative examples of BsSMC-dependent bridging of adjacent SYTOX Orange-labelled flow-stretched DNAs in the absence of ATP. [BsSMC]=180 nM. Arrows indicate the DNAs of interest. Bridging was observed in 34 DNA pairs out of 85 pairs of closely spaced DNAs.

We found that BsSMC-mediated DNA compaction exhibited characteristics of both DNA bending and bridging (see Fig. 3a and Supplementary Fig. 3a,b for representative events). In contrast to DNA-bridging proteins, such as ParB (Spo0J), which exhibit strict end bias in our assay, quantum dots along DNAs began to move towards the tether point at the same time in the vast majority (>80%) of trajectories, similar to the DNA bender, HBsu. Unlike DNA benders, however, BsSMC typically compacted DNA all the way to the tether point, a behaviour consistent with BsSMC also forming higher-order bridging interactions (Fig. 3a; Supplementary Fig. 3b). In addition, we observed bridging directly in assays in which SYTOX Orange-labelled DNAs in close proximity were crosslinked by BsSMC in both the absence (Fig. 3b; Supplementary Fig. 3c) and presence of ATP (Supplementary Fig. 3d). Bridging events were not observed to occur in the absence of BsSMC, but occurred robustly (40%) in its presence on DNAs that were close enough to interact.

### ATP modestly stimulates BsSMC-mediated DNA compaction

To characterize the effect of ATP on the action of BsSMC, we measured DNA compaction kinetics as a function of BsSMC concentration in its presence and absence. To carefully quantify the kinetics of DNA compaction, we used a DNA substrate in which only the free end was labelled with a quantum dot. Using this approach, we could ensure that the DNA was full length and that the compaction kinetics were measured from the same position on each DNA. After tethering the quantum dot-labelled DNAs onto the flow cell surface, a fixed concentration of (unlabelled) wild-type (WT) BsSMC was flowed in with a very small concentration of fluorescent tracer dye either with or without ATP. The arrival of BsSMC at the flow cell surface was determined by the increase in background intensity due to the tracer dye. Plots of quantum dot position versus time revealed that DNA compaction did not always occur with a constant speed. It was commonly observed that the rate of compaction would suddenly change, often slowing down as the compacting DNA neared the tether point. Therefore, we calculated the initial compaction rate over the interval of the onset of compaction until the transition to a different compaction rate (Supplementary Fig. 4a). Compaction rate histograms were broad, but were well fit with a Gaussian curve (Supplementary Fig. 4b), from which the mean and s.e.m. of the compaction rate were calculated.

**Figure 4:**
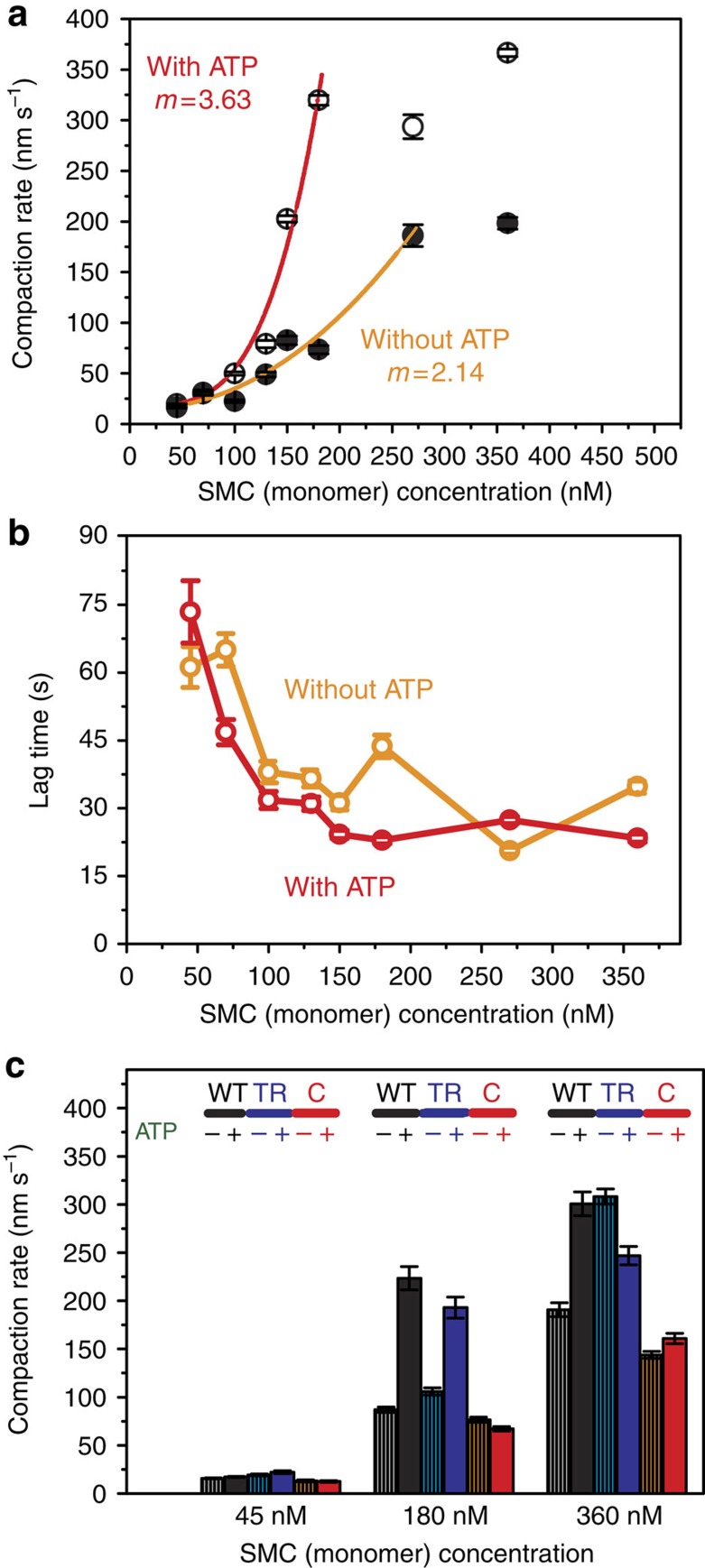
BsSMC compacts DNA in a weakly ATP-dependent manner. (**a**) Compaction rate versus BsSMC concentration. ‘*m*' was obtained from the least-square fitting of Rate ∝ [BsSMC]^*m*^ for data points before the compaction rate saturating. Closed and open circles represent data points (*n* ranged from 14 to 73) without and with ATP, respectively. Error bar: s.e.m. (**b**) Lag time versus BsSMC concentration (*n* ranged from 14 to 73). Error bar: s.e.m. (**c**) Compaction rates for wild-type (WT), transition-state (TR) and C-motif (C) mutants BsSMC both in the absence and presence of ATP (*n* ranged from 32 to 131). Error bar: s.e.m.

As shown in Fig. 4a, ATP weakly stimulates the DNA condensation activity of BsSMC. Consistent with the observation that BsSMC cluster formation does not require ATP, we found relatively robust DNA compaction in its absence. At saturating concentrations of BsSMC, we saw an approximately twofold increase in the compaction rate with ATP. Importantly, the compaction rate increases with BsSMC concentration, following a power law with exponents of 3.6 and 2.1 in the presence and absence of ATP, respectively (Fig. 4a). This difference in scaling suggests that ATP does enhance cooperative interactions between BsSMC dimers, consistent with our observations that BsSMCs localize into clusters and that they are able to bridge DNAs. In accordance with the observation that preincubation of ScpAB with BsSMC decreased the number and brightness of SMC clusters, we found it also decreased the DNA compaction rate, providing further support to the hypothesis that the non-SMC subunits regulate BsSMC activity but do not change it qualitatively (Supplementary Fig. 4c).

The initiation of DNA compaction was not coincident with the arrival of protein into the flow cell as has been observed for MukB^41^. Instead, we observed a nonzero lag time even at high BsSMC concentrations (Fig. 4b). The existence of the lag time further suggests that BsSMCs interact cooperatively to condense DNA. Using the end-labelled DNA, we quantified the fraction of DNA compaction that was 99.0±2.2% of the full length of DNA without ATP, and 103.7±1.3% with ATP; mean±s.e.m. (see Methods). As observed in the DNA motion capture experiments, BsSMC was able to compact DNA to the tether point consistent with the formation of higher-order protein–DNA interactions.

### Two ATPase mutants of BsSMC disrupt DNA compaction

To further explore how the ATPase cycle of BsSMC is coupled with its DNA condensation activity, we expressed and purified two SMC ATPase mutants (Supplementary Fig. 4d), and measured their DNA compaction rates. The C-motif mutant (S1090R, BsSMC_C_) that inhibits head–head engagement and thus slows ATP hydrolysis (Fig. 1a)^11^,^51^ was still able to condense DNA with compaction rates (both with and without ATP) comparable to WT BsSMC in the absence of ATP (Fig. 4c). Notably, in the presence of ATP, the compaction rate of BsSMC_C_ is much lower than that of WT BsSMC (*P*<5 × 10^−12^ by *t*-test at both 180 and 360 nM; Fig. 4c). These results demonstrate that ATP-assisted proper head–head engagement is required to most efficiently condense flow-stretched DNAs. Inclusion of ScpA and ScpB reduced the compaction rate of BsSMC_C_ by approximately fivefold (*P*<5 × 10^−15^ by *t*-test, Supplementary Fig. 4e). Interestingly, a similar fold change was observed for the WT BsSMC (compare Supplementary Fig. 4c,e).

Next, we examined a BsSMC transition-state mutant (E1118Q, BsSMC_TR_), which slows down ATP hydrolysis, thereby increasing the duration of head engagement^11^,^20^. In the presence of ATP, BsSMC_TR_ compacts DNA more slowly than the WT at saturating SMC concentrations (*P*<5 × 10^−5^ by *t*-test at 360 nM; Fig. 4c), indicating that head–head opening that occurs upon ATP hydrolysis is also important for efficient compaction. Supporting the notion that the opening of the ATPase heads can limit the rate at which DNAs are condensed, we observed that adding ATPγS to the flow cell instead of ATP resulted in an approximately twofold reduction in the compaction rate (Supplementary Fig. 4f). Surprisingly, in the absence of ATP, the compaction rate of BsSMC_TR_ is higher than that of WT (*P*<5 × 10^−5^ by *t*-test for both 180 and 360 nM concentrations; Fig. 4c), contrary to our expectation that those rates would be identical. The origin of this effect remains unclear but could result from: (1) the E1118Q mutation stabilizing DNA binding by BsSMC_TR_ or (2) conformational changes within the ATPase due to the point mutation that could facilitate compaction.

### Hinge/coiled-coil domain condenses DNA by bending

**Figure 5:**
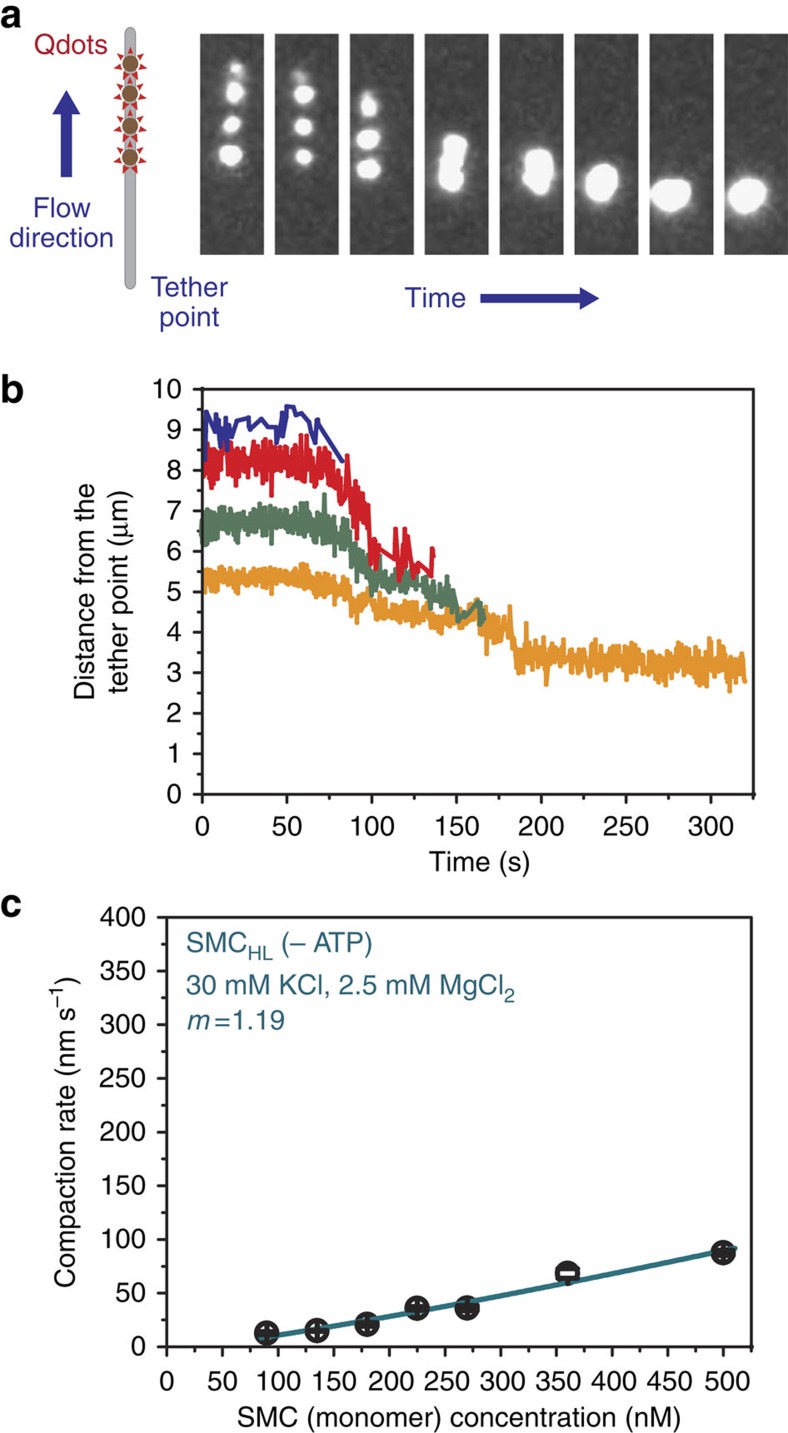
BsSMC_HL_ condenses DNA in a non-cooperative manner. (**a**) Snapshots from a DNA motion capture experiment showing DNA compaction by BsSMC_HL_. (**b**) Trajectory of the compaction shown in **a**. The position of each quantum dot is shown by a different coloured line. (**c**) Compaction rate versus BsSMC_HL_ concentration (*n* ranged from 18 to 42) was fit to the power-law expression rate∝[BsSMC]^*m*^. Error bar: s.e.m.

Blocking ATPase head engagement (BsSMC_C_) or slowing ATP hydrolysis (BsSMC_TR_) altered the kinetics of DNA compaction by BsSMC but did not completely eliminate DNA compaction activity. To further address the role of the ATPase head domain in DNA compaction, we purified a headless BsSMC construct comprised of residues 160–1037 (BsSMC_HL_) and used it in DNA motion capture experiments. To our surprise, BsSMC_HL_ was capable of globally compacting DNA (Fig. 5a,b). Although the head domain is dispensable for DNA condensation, its absence severely affected compaction kinetics. At an SMC concentration of 360 nM without ATP, the average compaction rate for WT BsSMC is 190.7 nm s^−1^, while for BsSMC_HL_ it is only 12.9 nm s^−1^. Even at a concentration of 1,000 nM BsSMC_HL_, the compaction rate is only 34.6 nm s^−1^. In addition, BsSMC_HL_ only compacted DNA by ∼73% of the flow-stretched DNA length as compared with 100% for the WT.

To characterize the DNA compaction kinetics of BsSMC_HL_, we reduced the salt concentration of the buffer from 100 mM KCl to 30 mM, while keeping the MgCl_2_ concentration at 2.5 mM. This substantially increased the rate of compaction allowing us to investigate how the compaction rate varied over a large protein concentration range (Fig. 5c; Supplementary Fig. 5a,b). In contrast to the WT BsSMC that showed increasing cooperativity in the presence of ATP, the compaction rate for BsSMC_HL_ increased linearly (power of 1.19) with increasing concentration. Collectively, these results suggest that each BsSMC_HL_ compacts DNA by itself, likely by bending DNA, and that the ATPase head domains are mainly responsible for cooperative bridging interactions.

## Discussion

**Figure 6:**
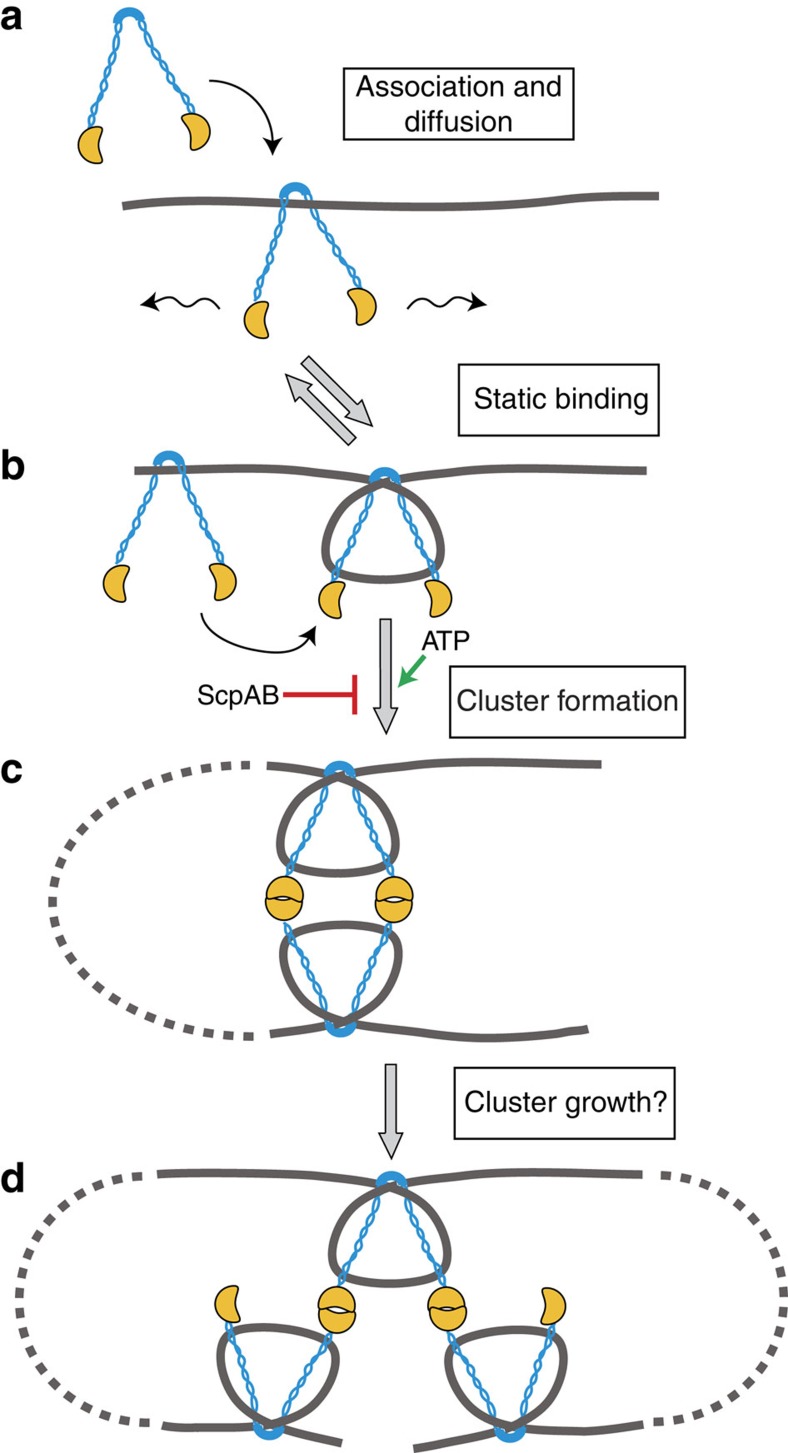
Working model of BsSMC loading and assembly into clusters. (**a**) BsSMC associates with DNA and can undergo 1D diffusion. (**b**) Diffusing BsSMC molecules transiently localize likely due to bending or wrapping of DNA around their hinge/coiled-coil arms. (**c**) Cooperative interactions between BsSMC molecules, predominately on the same DNA strand, trap DNA loops. ATP stimulates these interactions while the presence of ScpAB attenuates cluster formation. (**d**) Repeated cycles of ATP hydrolysis resulting in opening of the ATPase head domains may enable the formation of a network of interacting BsSMC molecules.

Genetic studies have clearly established that the bacterial SMC plays a critical role in chromosome organization, while biochemical experiments have demonstrated that SMC family members possess a number of DNA-modifying activities, including DNA condensation, bridging and supercoiling^35^,^36^,^38^,^39^,^40^,^41^. Yet, it remains unclear how these SMC-mediated changes in DNA contribute to chromosome structure. To better understand how SMC is loaded and assembled on DNA, we combined DNA flow stretching with the single-molecule imaging of fluorescently labelled BsSMC molecules. We demonstrated that BsSMC has a number of distinct interactions with DNA that evolve as the BsSMC complex assembles, providing a more complete picture of how BsSMC interacts with DNA and performs its function (Fig. 6).

Upon DNA binding, we found that BsSMC dimers switch between periods of 1D diffusion and static association (Fig. 6a,b). BsSMC diffusion was only weakly biased in the direction of flow, and substantially lower than the estimated fluid velocity at the height of the DNA (∼40 μm s^−1^; see Methods)^52^; an SMC ring moving at these velocities would traverse the whole length of flow-stretched DNA in under a second compared with the tens of seconds we typically observed. These data suggest that BsSMC must make relatively significant contact with DNA in the diffusing state even if BsSMC adopts a ‘ring-like' conformation.

Our direct observations of BsSMCs sliding along DNA support proposals that 1D diffusion of SMC family members might help to distribute them along the chromosome. Chromatin immunoprecipitation experiments in *Saccharomyces cerevisiae* demonstrated that cohesin, and to a much lesser extent condensin, can be found at distinct sites from its loader, Scc2–Scc4 (refs 53, 54). Actively transcribing RNA polymerase may push diffusing SMC family members ahead of it, resulting in SMC localization downstream of highly transcribed genes or at sites of convergent transcription^55^. Chromatin immunoprecipitation experiments in *B. subtilis* have similarly shown BsSMC enrichment at ribosomal genes^25^. Structural differences between condensin- and cohesin-like SMCs may bias how frequently they are in the diffusing versus statically bound state, thus influencing their ability to spread from their sites of loading.

What causes BsSMC dimers to switch between 1D diffusion and static binding to DNA? One possibility is that a conformational transition within the hinge or coiled-coil arm domains changes SMC from an open to closed conformation that tightly grips DNA. Prior structural studies by electron microscopy and atomic force microscopy have shown that SMC family members can adopt an open configuration of the coiled-coil arms (‘open-V' structure) or the arms may lie quite close to each other in a ‘folded-rod' conformation^14^,^56^. These states likely have different affinities for DNA. However, recent biochemical and structural work suggests that the SMC hinge and coiled-coil arm domains undergo a conformational transition from the rod like to the more open configuration upon DNA binding^57^.

A second possibility, which is not necessarily mutually exclusive with the conformational transition model above, is that BsSMC may bend (or wrap) DNA around itself, arresting its 1D diffusion (Fig. 6b). Supporting a role for BsSMC in locally bending DNA, we found that WT BsSMC predominately compacts DNA globally, a pattern we have previously attributed to DNA-bending proteins^43^. In addition, BsSMC_HL_ is able to compact DNA in a non-cooperative manner, implying a direct role for the hinge/coiled-coil domain in DNA condensation. Such behaviour does not appear to be unique to the bacterial condensin, as magnetic tweezers experiments demonstrated that a headless construct of cohesin SMC1/3 could also compact DNA^39^. In line with a DNA-bending model, magnetic tweezers experiments with MukB and cohesin (SMC1/3) both showed a preference for certain types of DNA crossings; MukB was found to more readily bridge DNAs that were intertwined with positive twist, while positively supercoiled DNA was more readily condensed by SMC1/3 (refs 38, 39).

At increasing SMC concentrations, we observed that BsSMCs formed multiple clusters along stretched DNAs. We propose that the static association of BsSMC on DNA nucleates cluster formation, a suggestion that is supported by the observed lag time between protein arrival into the flow cell and subsequent cluster formation and initiation of DNA compaction. Further cluster growth may result from the direct recruitment of additional BsSMCs from solution or via their 1D diffusion along DNA.

BsSMC clusters act as local DNA condensation centres (Fig. 6c). Cluster formation occurred before measurable DNA compaction, while DNA motion capture experiments showed that DNAs compact simultaneously along their length and not just from the free DNA end. Prior single-molecule studies of SMC family members have similarly shown that they can compact DNA under weak forces although different SMCs possess functional differences. While ATPase activity is required for SMC function *in vivo*, *in vitro* experiments show that MukB, the SMC1/3 cohesin heterodimer and BsSMC (shown here) are able to condense DNA in its absence^39^,^40^,^41^. Only condensin I complex purified from *Xenopus laevis* egg extracts strictly required ATP for DNA compaction^40^.

What roles then does ATP play in the interaction of BsSMC with DNA? Prior biochemical work concluded that BsSMC initially associates with DNA through its hinge domain and that binding is independent of ATP^17^, consistent with proposals that SMC binding and SMC-mediated DNA compaction are decoupled processes^38^,^58^. Supporting these suggestions, we find that the presence of ATP does not lead to statistically significant differences in the diffusive properties (diffusion distance, drift and diffusion coefficient) of BsSMC on DNA. Instead, ATP seems to facilitate higher-order interactions between BsSMCs likely mediated through the ATPase head domains. We find that the DNA compaction rate as a function of BsSMC concentration is more cooperative in the presence of ATP, although ATP is not required for DNA compaction. Therefore, ATP likely biases BsSMC to adopt conformations that facilitate DNA compaction, but even in its absence, BsSMC can sample these configurations due to thermal fluctuations.

In addition, we found that BsSMC mutants disrupting the ATPase cycle lower the compaction rate in the presence of ATP. The C-motif mutation, BsSMC_C_, is believed to inhibit head–head engagement^11^,^12^. Yet, we found that BsSMC_C_ could compact DNA in an ATP-independent manner, at rates identical to the WT in the absence of ATP. Again, we speculate that even with the C-motif mutation the ATPase heads are able to weakly or transiently associate with each other but this conformation cannot be stabilized by ATP binding. Decreasing the rate of ATP hydrolysis with the transition-state mutant, BsSMC_TR_, decreased the rate of DNA compaction, suggesting that potentially multiple cycles of ATP hydrolysis occur before the formation of productive interactions with neighbouring SMCs. Introduction of the C-motif or transition-state mutation into *B. subtilis* leads to growth defects on nutrient rich medium similar to *smc* null cells and results in impaired loading of SMC complexes^59^. Strains containing the transition-state mutation in *Caulobacter crescentus*^60^ are impaired in chromosome segregation, while in *Escherichia coli*^61^ the transition-state mutation leads to a marked stabilization of MukB and its binding partners MukE and MukF (MukBEF) on the chromosome. Taken together, these ATPase mutants demonstrate that the interaction of the ATPase heads is finely tuned; either weakening (BsSMC_C_) or strengthening (BsSMC_TR_) the association of the ATPase heads decreases the rate of productive complex formation.

Within bacterial cells, SMCs localize into foci near the origin region and require ScpAB, ParB and *parS* for formation^22^,^25^,^26^,^27^,^47^. Our observations that BsSMC cluster number and the number of SMC dimers within clusters decrease when ScpAB are present add to growing biochemical evidence that ScpAB negatively regulates DNA binding by SMCs^20^,^62^. On the other hand, ParB likely targets and facilitates SMC loading by binding to origin-proximal *parS* sites and recruiting SMC either through direct physical interactions or by stabilizing loop domains that SMC may preferentially bind to^25^,^26^,^43^,^59^.

The exact nature of SMC foci in bacteria remains unclear; diffraction-limited foci may result from SMCs acting together in higher-order structures or may be due to a tight packing of non-interacting SMCs. Supporting a role for higher-order SMC interactions, single-molecule imaging studies in *E. coli* concluded that a dimer of the dimeric MukBEF serves as the minimal functional unit in cells with 8–10 of these complexes clustering in ∼50-nm foci that condense DNA^61^. Chromosome conformation capture experiments in *B. subtilis* showed that BsSMC is responsible for forming interarm contacts along the chromosome, while origin-proximal BsSMCs ‘zip' up DNA flanking *parS* sites^63^,^64^.

The mechanisms by which BsSMCs condense the chromosome near the origin, are distributed along the chromosome at distant sites from their loading and establish interactions between the chromosome arms have yet to be fully elucidated. Our data demonstrating that BsSMC can diffuse on and bridge DNA segments present possible answers to the latter two questions. Regarding how BsSMC molecules might structure the origin, we show that *in vitro* BsSMC clusters compact DNA cooperatively through interactions within the ATPase head domains. Yet, our studies are not able to resolve whether this is carried out by a dimer of BsSMC dimers or by a more complicated network of interactions (Fig. 6d) nor do we know whether these clusters are functionally equivalent to SMC foci *in vivo*. Exactly how DNA is condensed within these *in vitro* BsSMC clusters will require further study. Our proposal that BsSMC molecules can both bend and bridge DNAs suggests the following picture: the bending of DNA around an individual BsSMC leads to the formation of a supercoiled loop, while further bridging interactions between BsSMC molecules would bring these supercoiled domains together forming higher-order DNA structures. In this way, BsSMCs act cooperatively to both condense DNA and organize it within supercoiled domains.

## Methods

All protein concentrations were denoted in terms of monomer throughout this article.

### Plasmid preparations

WT BsSMC plasmid with the BsSMC gene subcloned into the NdeI and XhoI sites of the pET-24(+) vector was a generous gift from David Rudner. The plasmid was designed such that the C terminus of the BsSMC is immediately followed by a cloning scar (Leu–Glu) and six histidines.

To generate plasmids for BsSMC_C_ (E1118Q) and BsSMC_TR_ (S1090R), site-specific mutagenesis was performed as follows: the sequences of primers (purchased from IDT) are shown in the Table 1 (TR-Forward, TR-Reverse, C-Forward and C-Reverse). Excess forward and reverse primers were mixed with the WT BsSMC plasmid, dNTPs (final concentration of 0.2 mM each), 1/8 volume of *PfuUltra* High-Fidelity DNA Polymerase AD (Agilent) and vendor-supplied buffer. After 20 rounds of PCR, the reactions were digested with DpnI (NEB) for 3 h at 37 °C and transformed into a cloning strain of *E. coli.* The entire coding sequence of each clone was verified by sequencing.

For the BsSMC_HL_ (160–1,037) plasmid, a fragment of BsSMC corresponding to amino acids 160–1,037 was PCR-amplified with sense (named as HL-Sense, for sequence see Table 1) and antisense (named as HL-Antisense, for sequence see Table 1) primers using KOD Hot Start Master Mix DNA Polymerase (EMD Millipore), digested with NdeI and XhoI and ligated into the same sites in pET-24(+). Clones were checked by DNA sequencing.

Plasmids for ScpA-His_6_, where the ScpA-coding sequence was subcloned into pET-24b(+) between the NheI and HindIII sites, and for His_6_-ScpB, where the ScpB-coding sequence was subcloned into pRsetA between the NheI and XhoI sites, were kind gifts from Xindan Wang in the Rudner laboratory.

### BsSMC protein expression and purification

Rosetta2(DE3)pLysS competent cells (EMD Millipore) were transformed with BsSMC plasmid (WT or BsSMC_c_ or BsSMC_TR_) and plated on LB agar (EMD Millipore) with 50 μg ml^−1^ kanamycin and 20 μg ml^−1^ chloramphenicol. Cells from a single colony were grown in small cultures in LB with kanamycin and chloramphenicol (2–3 ml for several hours followed by 100 ml overnight). The next day, this starter culture was used to inoculate 1.5 l LB with 40 μg ml^−1^ kanamycin and grown at 37 °C to an OD_600_ of 0.5–0.6. Expression was induced with 200 μM isopropyl-β-D-thiogalactoside (Gold Biotechnology) at 26–27 °C for 4 h. The culture was centrifuged at 5,000*g* (Sorvall RC 5C Plus Superspeed) at 4 °C for 10 min, and the pellets were resuspended with lysis buffer (50 mM Na-phosphate, pH 8.0, 10% glycerol, 300 mM NaCl, 10 mM imidazole, 0.5 mM phenylmethylsulfonyl fluoride (PMSF)). Two rounds of freeze-thaw were performed, and 0.25 mg ml^−1^ lysozyme and an additional 0.5 mg ml^−1^ PMSF were added. After incubating 30 min on ice, the mixture was sonicated. Immediately, 2-mercaptoethanol (βME) was added to a final concentration of 5 mM, and the lysates were centrifuged at 11,000*g* for 30 min followed by 22,000*g* for 30 min (Sorvall SS-34 rotor) at 4 °C. The supernatant was mixed with ∼7 ml Ni-NTA agarose resin (washed with lysis buffer) and one tablet of mini protease inhibitor cocktail (Roche), and then incubated for 1 h at 4 °C. The mixture was poured into a column and washed with 10 column volumes (70 ml) of wash-1 buffer (50 mM Na-phosphate, pH 6.1, 10% glycerol, 300 mM NaCl, 50 mM imidazole, 0.2 mM PMSF, 5 mM βME) and 2 column volumes of wash-2 buffer (50 mM Na-phosphate, pH 7.5, 10% glycerol, 300 mM NaCl, 60 mM imidazole, 0.2 mM PMSF, 5 mM βME). BsSMC protein was eluted by applying a gradient of imidazole (130–500 mM) in elution buffer (50 mM Na-phosphate, pH 7.5, 10% glycerol, 300 mM NaCl, various concentration of imidazole, 5 mM βME). The eluates were run on an SDS–polyacrylamide gel electrophoresis (SDS–PAGE) gel, which was stained with InstantBlue (Expedeon). Peak fractions were pooled and concentrated to ∼3 ml by centrifugation in a 100-kDa MWCO concentrator (EMD Millipore) at 5000g in a fixed-angle rotor (Eppendorf) at 4 °C. The protein was dialysed overnight against gel-filtration buffer (20 mM HEPES, pH 7.8, 10% glycerol, 1 mM EDTA, 50 mM KCl, 0.2 mM PMSF, 5 mM βME, 2 tablets (per 1 litre) of mini protease inhibitor cocktail). The protein was further purified by gel filtration using an ÄKTA Prime Plus FPLC and a Superdex 200 column (HiLoad 16/60, prep grade, GE Healthcare; FPLC buffer: 20 mM HEPES, pH 7.8, 10% glycerol, 1 mM EDTA, 50 mM KCl, 1 mM βME). Elution fractions were analysed by SDS–PAGE and InstantBlue staining. Peak fractions were pooled and concentrated as before. Absorbance at 280 nm was measured with a NanoDrop spectrophotometer (Thermo Scientific) and used to calculate the final protein concentration based on a calculated extinction coefficient of 51,230 M^−1^ cm^−1^ and molecular mass of 135.51 kDa. The purified protein was aliquoted, flash-frozen in liquid nitrogen and stored at −80 °C.

BsSMC_HL_ was purified essentially as described above but using a 30-kDa MWCO (EMD Millipore) concentrator due to its lower molecular mass.

### ScpAB protein expression and purification

For the purification of ScpA and ScpB, BL21(DE3)pLysS competent cells were grown in LB medium with appropriate antibiotics (for ScpA: 50 μg ml^−1^ kanamycin and 20 μg ml^−1^ chloramphenicol, for ScpB: 100 μg ml^−1^ ampicillin and 20 μg ml^−1^ chloramphenicol). Protein expression was induced by adding 300 μM isopropyl-β-D-thiogalactoside at 26–27 °C for 4 h. After sonication, the lysates were centrifuged twice at 22,000*g* for 30 min, and the supernatant was incubated with 6 ml of Ni-NTA agarose (Qiagen) for 1 h at 4 °C. The Ni-NTA agarose slurry was then poured into a disposable column and washed with 3 column volumes (24 ml) of wash-1 buffer (50 mM Na-phosphate, pH 6.1, 10% glycerol, 300 mM NaCl, 50 mM imidazole, 1 mM PMSF, 5 mM βME), 2 column volumes of wash-2 buffer (50 mM Na-phosphate, pH 7.5, 10% glycerol, 300 mM NaCl, 50 mM imidazole, 1 mM PMSF, 5 mM βME) and ∼1.7 column volumes of wash-3 buffer (50 mM Na-phosphate, pH 7.5, 10% glycerol, 1000, mM NaCl, 50 mM imidazole, 1 mM PMSF, 5 mM βME). Protein was eluted with elution buffer (50 mM Na-phosphate, pH 7.5, 10% glycerol, 300 mM NaCl, various concentration of imidazole, 5 mM βME). Peak elution fractions were pooled and dialysed overnight against 20 mM HEPES, pH 7.7, 10% glycerol, 50 mM KCl, 1 mM EDTA and 5 mM βME. The dialysate was concentrated using a 5-kDa MWCO Vivaspin concentrator (GE Healthcare), aliquoted, flash-frozen in liquid nitrogen and stored at −80 °C.

### Labelling BsSMC with fluorescent dye

BsSMC stock solution was supplemented with a 1.1-fold excess of Cy3 NHS ester (GE Healthcare) dissolved in dimethylsulphoxide and left overnight at 4 °C. Free dye was removed using Micro Bio-Spin P-30 Gel Columns (Bio-Rad). We found that this step was crucial, since using a centrifugal concentrator to remove free dye abolished the protein's activity. After running the sample on an SDS–PAGE gel, the labelling was confirmed using a Typhoon imager (GE Healthcare), and the labelling efficiency was calculated by measuring absorbance at both 280 and 550 nm using a NanoDrop spectrophotometer (Thermo Scientific). The labelling efficiency was around 50% (approximately one Cy3 dye per BsSMC dimer).

The compaction rate with unlabelled WT BsSMC (Fig. 4a) was used to gauge activity of the labelled WT BsSMC. When a high concentration of Cy3-labelled BsSMC was flowed in, a spectrally distinct quantum dot attached at the end of the flow-stretched DNA was imaged onto the other half of an electron multiplying charge coupled device (EMCCD) camera. The position of the quantum dot was determined by Gaussian fitting and used to determine the compaction rate. The rate of compaction by labelled BsSMC was comparable to that by unlabelled BsSMC, indicating that labelling does not disrupt the DNA-compacting activity of the protein.

### Biotinylation and quantum dot labelling of λ DNA

To label one end of a genomic bacteriophage λ-DNA with a biotin^43^, we took an advantage of the fact that both ends have 12-base 5′-overhang called a *cos* site. A short biotin-oligo (named as Lambda-BL1Biotin, IDT, see Table 1 for the sequence) was phosphorylated by T4 polynucleotide kinase (PNK) at 37 °C for 1 h. The λ-DNA (NEB) was mixed with a 10-fold excess of the phosphorylated short Lambda-BL1Biotin-oligo and incubated at 65 °C for 10 min. Then, it was annealed by slow cooling to room temperature, ligated using T4 ligase (NEB) and stored at 4 °C until use. For the DNA motion capture assay, labelling of DNA with quantum dots were done just before each single-molecule experiments as follows: catalytically inactive His_6_-EcoRI_E111Q_ (a gift from Thomas Graham. See Graham *et al.*^43^) was incubated for 30 min at room temperature with anti-His antibody-conjugated quantum dots (antibody: AbD Serotec, MCA1396; quantum dot: Invitrogen, Antibody conjugation kit) in 20 mM Tris, pH 7.5, 100 mM NaCl, and 0.1 mg ml^−1^ bovine serum albumin. Biotinylated λ-DNA was added, and the mixture was allowed to incubate for 30 min at room temperature. This DNA stock was used in DNA motion capture assay and diffusion/static association experiments.

For modifying one end of λ-DNA with biotin and labelling the other end with a single quantum dot, a slightly different protocol was applied. First, both a digoxigenin-oligo (named as Lambda-Dig, IDT, see Table 1 for the sequence) and a biotin-oligo (named as Lambda-BL2Biotin, IDT, see Table 1 for the sequence) were phosphorylated by T4 polynucleotide kinase (PNK) at 37 °C for 1 h. A mixture of λ-DNA and the phosphorylated short Lambda-Dig-oligo (15-fold excess) in 1 × T4 DNA ligase buffer (NEB) was heated to 65 °C for 10 min and annealed by slow cooling to room temperature. T4 DNA ligase and 0.8 mM ATP were added, and the reaction was allowed to proceed at room temperature for 2 h. After that, a 60-fold excess (compared with λ-DNA) of the phosphorylated short Lambda-BL2Biotin-oligo was added, and the mixture was heated at 45 °C for 30 min and then slowly cooled to room temperature. Again, T4 ligase and 0.8 mM ATP were added, and the reaction was allowed to proceed at room temperature for 2 h. Since the sequence of the Lambda-Dig-oligo is complementary to that of the Lambda-BL2Biotin-oligo, removing excess oligos is crucial to prevent tethering of anti-digoxigenin quantum dots to short oligo duplexes on the surface. For that purpose, the labelled DNA was separated from free short oligos by electrophoresis on a 0.4% agarose gel at low voltage (15–25 V) overnight. The band containing λ DNA was excised, and the DNA was recovered by electroelution into a dialysis bag followed by ethanol precipitation. Labelling of biotin-λ-dig DNA was performed each time before the start of single-molecule experiment by mixing the prepared biotin-λ-dig DNA with an excess amount (about 20-folds) of anti-digoxigenin antibody-conjugated quantum dots (antibody: Roche Life Science, 11 214 667 001; quantum dot: Invitrogen, Antibody conjugation kit).

### Single-molecule flow-stretching assay

Coverglasses were cleaned by extensive sonication in ethanol and 1 M potassium hydroxide, treated with (3-aminopropyl)triethoxysilane (Sigma-Aldrich) and surface-passivated using a 25:1 mixture of mPEG-SVA and biotin-PEG-SVA (Laysan Bio; or 25:2.5:1 mixture of mPEG-SVA, SVA-PEG-SVA and biotin-PEG for cluster formation experiments). Flow cells were constructed with the PEGylated coverglasses and quartz slides (with holes) where double-sided tape was sandwiched between them. PE60 tubing (VWR) was inserted into the holes as an inlet and an outlet. The inlet tubing was placed in the buffer/sample solution, and the outlet tubing was connected to an air spring and syringe pump (Harvard Apparatus). All experiments were performed on an inverted total internal reflection fluorescence microscope (IX71, Olympus). Expanded (and collimated) 532-nm laser light (Coherent) was focused at the back focal plane of the objective with a near-total internal reflection critical incidence angle, and the fluorescence emission signal was imaged on an EMCCD camera (Hamamatsu). The exposure time for compaction experiments was 100 ms, and data were taken every 200 ms. For the experiments with low concentration of BsSMC, data were taken every 100 ms.

Neutravidin (0.25 mg ml^−1^; Thermo Scientific) was first added into the flow cell. After 5–10 min, excess neutravidin was washed away by flowing in EBB buffer (10 mM Tris, pH 8.0, 150 mM NaCl, 10 mM MgCl_2_, 0.2 mg ml^−1^ bovine serum albumin). Quantum dot-labelled biotinylated λ-DNA was flowed in at a rate of ∼0.02 ml min^−1^, and unbound excess DNA was washed away by flowing in SMC buffer (20 mM HEPES, pH 7.6, 100 mM KCl, 2.5 mM MgCl_2_) for the most experiments. SMC_30_ buffer with 20 mM HEPES, pH 7.6, 30 mM KCl, 2.5 mM MgCl_2_ was used for some of experiments with BsSMC_HL_ as mentioned in the main text. Finally, BsSMC in SMC buffer (or BsSMC_HL_ in SMC_30_ buffer) either with or without ATP was flowed in at a rate of 0.05 ml min^−1^, while emission signals from quantum dots were recorded with an EMCCD camera and Micro-Manager software^65^. As optional steps: (1) a movie was recorded, before flowing in BsSMCs, with no buffer flow (where average quantum dot position over time is the DNA tether point) followed by flowing buffer (where the DNA is stretched) to measure DNA length. The fraction of compaction was later calculated by dividing the length of compactions by the measured DNA length. (2) To determine at what time unlabelled BsSMC arrived in the flow cell, a small amount of a fluorescent dye (Cy3 or Alexa 555) was added in addition to BsSMC.

### Data analysis

A raw image file was opened up with ImageJ (NIH) to identify compaction events and coordinates of regions of interest. Then, custom-written MATLAB (MathWorks) codes were used to fit emission signals within each regions of interest to a Gaussian function to extract the coordinates of each quantum dot (Supplementary Softwares 1 and 2). Subsequent analyses, such as compaction rate calculation (Supplementary Software 3) or identification of the BsSMC arrival time (Supplementary Softwares 4 and 5), were also performed with custom-written MATLAB codes. Gaussian fitting of the compaction rate histogram (and also other histograms through this study) was done with Origin (Origin Lab).

To confirm that the Cy3 signals observed in BsSMC diffusion experiments corresponded to single BsSMC dimers, ratios of integrated signal from BsSMC-Cy3 on DNA (after background subtraction) to that from nearby nonspecifically surface-bound BsSMC-Cy3 were calculated. Prior studies demonstrated that BsSMC molecules exist almost exclusively as single dimers in solution^14^,^18^,^51^, therefore we assumed that surface stuck BsSMCs were single dimers. Given that our excitation conditions are near-total internal reflection, surface-bound molecules are brighter than those bound to DNA. We calculated a correction factor for this difference in excitation by comparing intensities of quantum dots labelled on DNA with those nonspecifically bound to the surface, the former had 68% of the brightness of the latter.

As the first step of diffusion coefficient calculation, positions of Cy3 were determined by Gaussian fitting. Excluding static parts as explained in the main text, mobile parts were concatenated as if they were segments of a single trajectory, and the average velocity (end-to-end distance divided by total time duration) was calculated, following Tafvizi *et al*^44^. If there were no bias from the flow, the net displacement over time would be close to zero. In our case, we observed a nonzero average drift velocity (Supplementary Fig. 1h), which we subtracted from every individual mobile trajectory. The corrected Cy3 coordinates were used to calculate the MSD using the equation below:


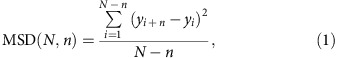


where *y* is a position coordinate along the DNA and *N* denotes the total number of data points (steps) in each trajectory. *n* is spanning from 1 to *N*. Diffusion coefficient *D* was calculated from linear line fitting using MSD(*N*, *n*)=2*Dn*Δ*t* with the following restrictions applied: (1) only *n*=2–15 were used, where the lower limit was intended to exclude potential nonlinear behaviour due to DNA fluctuations and the upper limit was determined to remove bounded motion from the short trajectories. (2) Only events with a Pearson correlation coefficient (calculated from CORREL function in Microsoft Excel) >0.90 for ‘MSD(*N*, *n*) versus *n*Δ*t*' graph were used in the diffusion coefficient calculation shown in Fig. 1g. Over 70% of trajectories met this criterion. These two restrictions were also used in the drift velocity histogram in Supplementary Fig. 1h.

To verify that data from BsSMC diffusion experiments were normally distributed, Kolmogorov–Smirnov tests were carried out using the Matlab built-in function (kstest). Excluding one apparent outlier that appeared in the diffusion coefficient histogram (with ATP) whose value was over 1.4 μm^2^ s^−1^, the distributions for net displacement, diffusion coefficient and drift were all found to be normally distributed at the 5% significance level, allowing for statistical comparisons of the distributions in the presence and absence of ATP. It was first determined using Microsoft Excel whether the variances of two data sets were the same (F-Test), and the appropriate *t*-test (assuming equal or unequal variances) was then performed. As another statistical comparison between the ATP and no ATP distributions, 95% confidence intervals were determined from 2,000 bootstrap distributions using the built-in Matlab functions ‘bootstrap' and ‘bootci'.

The mean height of the DNA from a PEGylated coverglass is about 0.2 μm (ref. 52), and the width and thickness (height) of our flow cell channel are 1.8 and 0.12 mm, respectively. Under the 0.050 ml min^−1^ flow condition that we used, the average fluid velocity *v*_avg_ is calculated to be 3,858 μm s^−1^. Using the equations below^66^, we calculated the fluid velocity at *y*=0.2 μm.


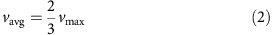



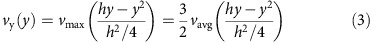


(*h*: height, 0.12 mm).

In our condition, *v*_y_ (at *y*=0.2 μm) is around 39 μm s^−1^. Even at a height of 0.05 μm, the fluid velocity *v*_y_ (at *y*=0.05 μm) is around 10 μm s^−1^, which is much faster than diffusion speed.

For DNA motion capture assays, simultaneous tracking of multiple quantum dots was performed using U-track software (Harvard University), following Aguet *et al.*^67^ and Jaqaman *et al.*^68^ More specifically, ‘Point Source Detection' section in U-track was used.

The number of BsSMC clusters formed per flow-stretched DNA (labelled with spectrally distinct quantum dot 705 at its end) was visually counted 5 min (250 μl) after flow of BsSMC-Cy3 and 1 mM ATP into the flow cell was initiated. These experiments were conducted both in the presence and absence of ScpA (fourfold higher concentration) and ScpB (eightfold higher concentration). The brightness of BsSMC clusters on DNA was compared with individual BsSMC molecules nonspecifically stuck to the surface in order to estimate the number of BsSMCs per cluster. These nonspecifically bound molecules were imaged at the end of the experiment after excess unbound BsSMCs were washed from the flow cell with buffer that did not contain any proteins. As described above, the intensity of DNA-bound BsSMCs was corrected for the difference in excitation between the surface and DNA.

BsSMC cluster positions on DNA were determined after the DNA was allowed to compact by 2 μm as determined by tracking the quantum dot 705 that labelled the end of DNA. Cluster positions were normalized with the tether point of DNA being set to zero and the end of DNA after compaction was set to one.

## Additional information

**How to cite this article:** Kim, H. & Loparo, J. J. Multistep assembly of DNA condensation clusters by SMC. *Nat. Commun.* 7:10200 doi: 10.1038/ncomms10200 (2016).

## Supplementary Material

Supplementary Information Supplementary Figures 1-5

Supplementary Software 1 This code fits emission signals for multiple regions of interest with Gaussian functions and reports their coordinates.

Supplementary Software 2 This code provides averaged data point trajectories obtained from qdot_track.m.

Supplementary Software 3 This allows the user to calculate compaction rates from the time versus position graph using a graphical interface.

Supplementary Software 4 This code calculates the time-course of background intensity for multiple regions of interest.

Supplementary Software 5 This code provides averaged intensity trajectories obtained from int_track.m.

## Figures and Tables

**Table 1:** Oligonucleotides used in this study.

Names	Sequences (5′ to 3′)
TR-Forward	g ttt tgc gtc ctt gac caa gta gag gct gcg ctc
TR-Reverse	gag cgc agc ctc tac ttg gtc aag gac gca aaa c
C-Forward	ac tta aac ctc ctg aga ggc gga gag cgt gcg
C-Reverse	cgc acg ctc tcc gcc tct cag gag gtt taa gt
HL-Sense	tctc catatg atctttgaagaagcggccg
HL-Antisense	gaga ctcgag gaatgtgtcgttaaagcgctt
Lambda-BL1Biotin	agg tcg ccg ccc/BiotinTEG/
Lambda-BL2Biotin	ggg cgg cga cct/BioTEG/
Lambda-Dig	agg tcg ccg ccc aaa aaa aaa aaa/Digoxigenin/

See Methods.
