# Supplementary material for: Multistep assembly of DNA condensation clusters by SMC
Source: Nat Commun. 2016 Jan 4;7:10200. doi: 10.1038/ncomms10200 (PMC4725763; doi:10.1038/ncomms10200)
Supplement: Supplementary Information — Supplementary Figures 1-5 [file ncomms10200-s1.pdf]

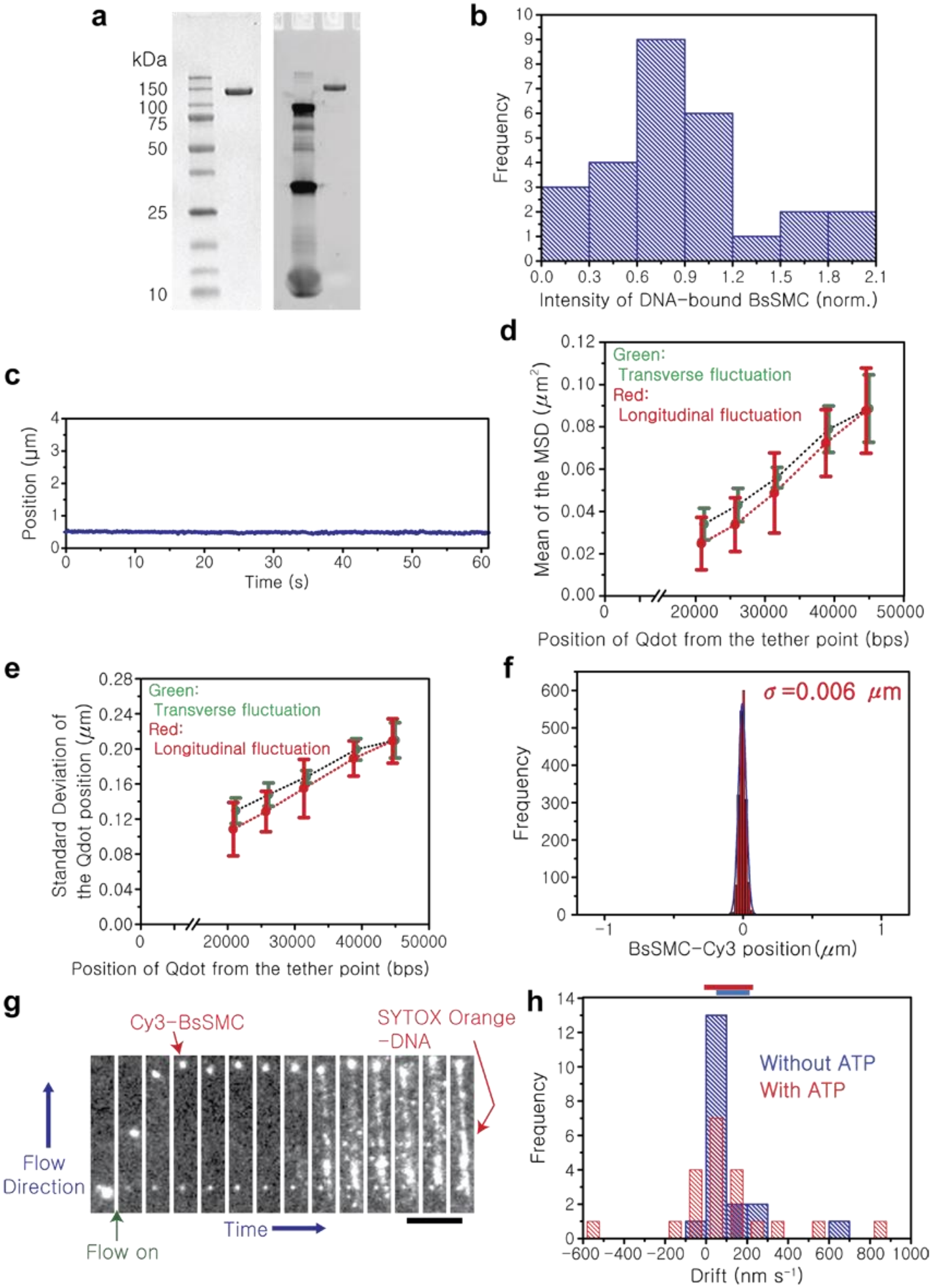


**Supplementary Figure 1: Two modes of low concentration of BsSMC on a DNA**

(**a**) Protein staining (left) and fluorescent imaging of Cy3 (right) confirm that BsSMC was labeled with Cy3 NHS-Ester. In each panel, the first and second lanes are protein ladder and protein, respectively.

(**b**) Intensity distribution of DNA bound BsSMCs normalized to the intensity of nonspecifically surface-bound BsSMCs. Intensity values of DNA-bound BsSMCs were corrected for differences in laser excitation between the surface and flow stretched DNAs under near total internal reflection imaging conditions. n=27.

(**c**) Position trajectory of a nonspecifically surface-bound BsSMC-Cy3 in the longitudinal direction.

(**d**) Mean values of MSD (mean-square-displacement) in the transverse and longitudinal directions for site-specifically bound-quantum dots on λ-DNA under laminar flow (n ranged from 4 to 7 DNAs). Quantum dot images were taken with 100 ms EMCCD exposure time. Fluctuations in both directions increase with increasing distance from the tether point. Error bars denote standard deviations.

(**e**) Standard deviations of the quantum dot positions also increase in both the transverse and longitudinal directions with increasing distance from the tether point (n ranged from 4 to 7 DNAs). Error bars denote standard deviations.

(**f**) Transverse position distribution of a nonspecifically surface-bound BsSMC-Cy3. $\sigma$ refers to the standard deviation of its position.

(**g**) BsSMC molecules that persisted until the end of the experiment were often found at or near the free end of the DNA as shown here. Buffer containing the intercalating dye SYTOX Orange was flowed into the channel after the experiment and DNAs were imaged. "Flow on" in the figure indicates when the SYTOX Orange-containing buffer began to flow in to the flow cell. Scale bar: 6 s

(**h**) Histogram of the drift velocity of mobile BsSMC spots on flow-stretched DNA. n=19 for blue bars, n=21 for red bars ($p>0.2$ by t-test). The horizontal blue (without ATP) and red (with ATP) lines on the top of the graph denote 95% confidence intervals calculated from bootstrapping analysis.

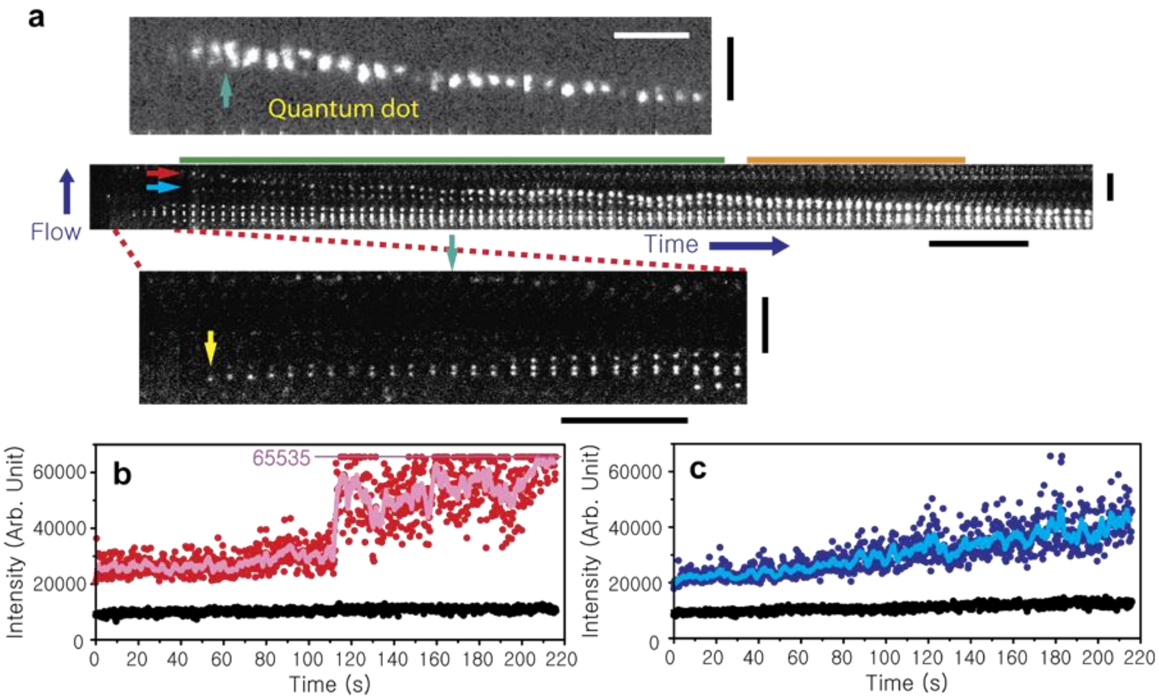


**Supplementary Figure 2: BsSMC cluster formation on a DNA**

(**a**) Another example of cluster formation in the absence of ATP. (The middle panel) The growth of clusters marked with the red and blue arrows over the time interval marked by the green bar are investigated in Supplementary Fig. 2b, c. The bottom panel shows a magnified view of the initial appearance of clusters. A yellow arrow indicates the appearance of the first visible cluster, and a green arrow denotes the onset of compaction, identified by tracking a DNA end-labeled quantum dot (shown in the top panel). Clusters occasionally merge, as demonstrated in the region marked with the orange bar (middle panel). Scale bars: (30 s, 5 μm), (40 s, 5 μm), and (5 s, 5 μm) from top to bottom panel

(**b** and **c**) Maximum intensities of clusters indicated by red and blue arrows increase over time. The intensity value of 65,535 indicates that the EMCCD pixel is saturated. Black dots indicate minimum intensities around clusters, showing that an increase in the background can not explain the changes in cluster intensity.

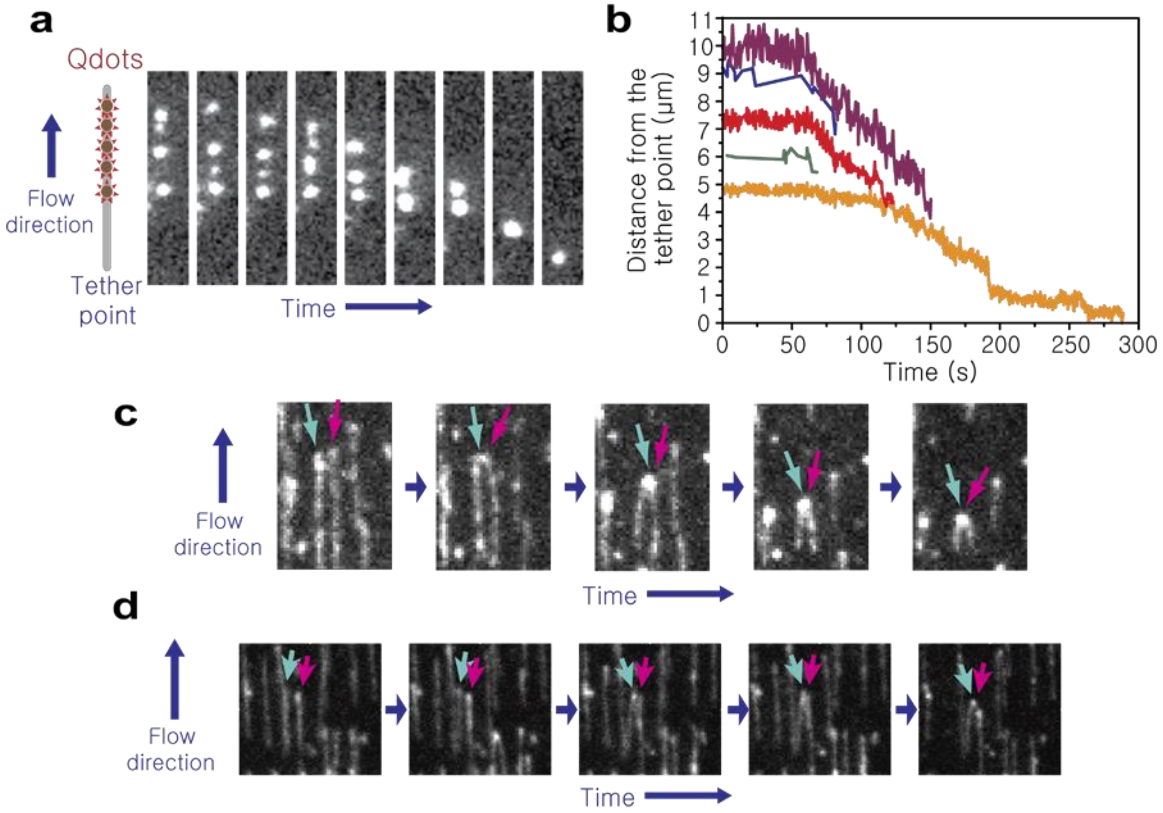


**Supplementary Figure 3: Cluster formation of BsSMC leads to DNA compaction**

(**a**) Snapshots of DNA motion capture assay with wild-type BsSMC in the absence of ATP.

(**b**) Trajectories of quantum dots shown in (**a**). Two of the five quantum dots (green and blue lines) blinked significantly over the course of the measurement.

(**c**) Another representative example of BsSMC-dependent bridging of adjacent SYTOX Orange-labeled flow-stretched DNAs in the absence of ATP. Two arrows indicate DNAs of interest. [SMC] = 180 nM.

(**d**) Two adjacent SYTOX Orange-labeled flow-stretched DNAs are bridged upon addition of BsSMC in the presence of 1 mM ATP. Two arrows indicate DNAs of interest. [SMC] = 120 nM.

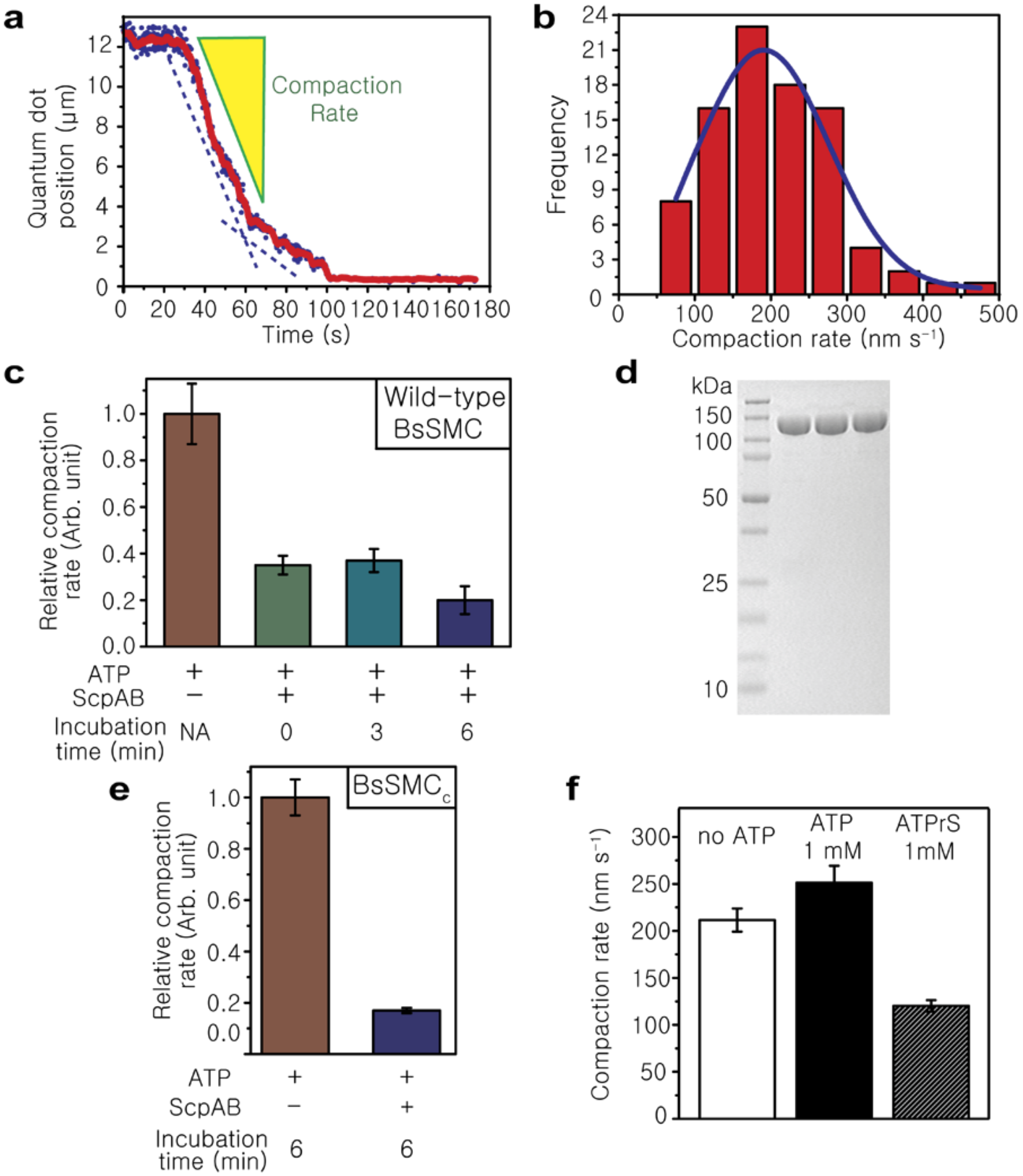


**Supplementary Figure 4: Quantification of end-labeled DNA compaction by wild-type BsSMC, $BsSMC_{TR}$, $BsSMC_C$**

(**a**) Representative compaction event by wild-type BsSMC (360 nM) in the absence of ATP. Two dotted lines indicate an abrupt change in the compaction rate. Only the initial compaction rate was considered in subsequent analyses.

(**b**) Histogram of initial compaction rate from experiments with same conditions (n=89). Histogram is well fit to a Gaussian curve. [wild-type BsSMC] = 360 nM without ATP.

(**c**) Relative compaction rate with 360 nM wild-type BsSMC (and 1 mM ATP) in the presence or absence of 1440 nM ScpA and 2880 nM ScpB. Incubation time indicates how long ScpAB, BsSMC, and ATP were incubated before flowing the mixture into the flow cell. Note that the actual incubation time is the sum of the incubation time indicated here and the time required for the mixture to travel from the sample tube into the flow

cell through the inlet tubing. Error bar lengths were calculated by error propagation. (n ranged from 16 to 31)

(**d**) SDS PAGE gel image for protein ladder, wild-type BsSMC, $BsSMC_{TR}$, and $BsSMC_{C}$. In order to compare compaction rates from these constructs accurately, they were purified to near-homogeneity.

(**e**) Relative compaction rate with 360 nM $BsSMC_c$ (and 1 mM ATP) in the presence (n=61) or absence (n=57) of 1440 nM ScpA and 2880 nM ScpB. $p<5x10^{-15}$ by t-test.

(**f**) Compaction rates of 360 nM wild-type BsSMC without ATP (n=41), with 1 mM ATP (n=32), with 1 mM ATPγS (n=65). Error bar: s.e.m.

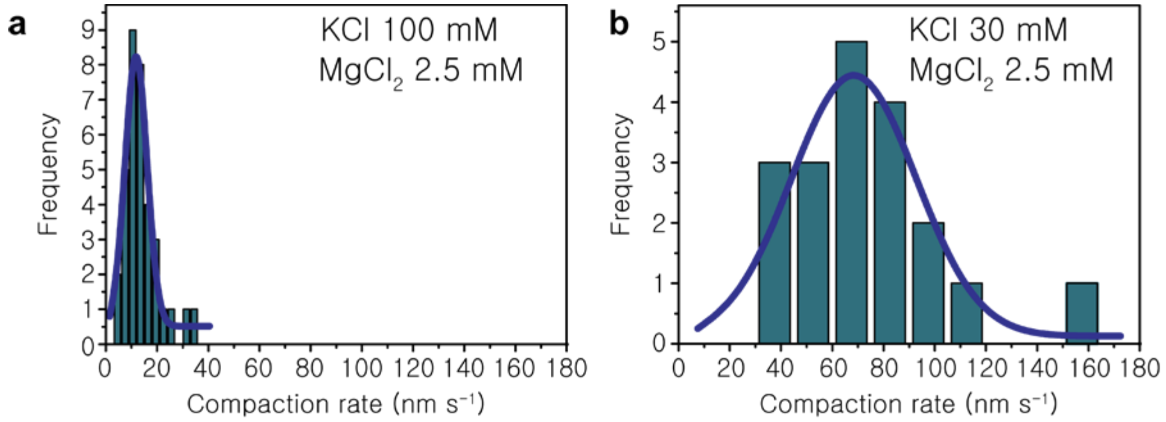


**Supplementary Figure 5: Compaction rate of $BsSMC_{HL}$ is dependent on salt concentration**

(**a**) Compaction rate of $BsSMC_{HL}$ (360 nM) under modest salt condition (n=35). This salt condition was used in other compaction experiments with wild-type BsSMC, $BsSMC_{TR}$, and $BsSMC_{C}$.

(**b**) Compaction rate of $BsSMC_{HL}$ (360 nM) under low salt condition (n=19)
